# Elevated Anxiety and Depression Scores are associated with reduced heart rate variability in women with polycystic ovary syndrome

**DOI:** 10.61622/rbgo/2026rbgo63

**Published:** 2026-08-20

**Authors:** Victor Barbosa Ribeiro, João Vitor Martins Bernal, Iris Palma Lopes Ribeiro, Gislaine Satyko Kogure, Stella Vieira Philbois, Rosana Maria dos Reis, Lucia Alves Silva Lara, Hugo Celso Dutra de Souza

**Affiliations:** 1 Instituto Federal de Educação, Ciência e Tecnologia da Paraíba João Pessoa PB Brazil Instituto Federal de Educação, Ciência e Tecnologia da Paraíba, João Pessoa, PB, Brazil.; 2 Universidade de São Paulo Faculdade de Medicina de Ribeirão Preto Departamento de Ginecologia e Obstetrícia Ribeirão Preto SP Brazil Departamento de Ginecologia e Obstetrícia, Faculdade de Medicina de Ribeirão Preto, Universidade de São Paulo, Ribeirão Preto, SP, Brazil.; 3 Universidade de São Paulo Faculdade de Medicina de Ribeirão Preto Departamento de Ciências da Saúde Ribeirão Preto SP Brazil Departamento de Ciências da Saúde Faculdade de Medicina de Ribeirão Preto, Universidade de São Paulo, Ribeirão Preto, SP, Brazil.

**Keywords:** Affective symptoms, Polycystic ovary syndrome, Body composition, Anxiety, Depression, Heart rate

## Abstract

**Objective:**

Polycystic ovary syndrome (PCOS) affects approximately 10% of women and can impact endocrine, metabolic, cardiovascular, and emotional parameters. This cross-sectional study aimed to investigate the relationship between cardiac autonomic modulation, assessed by heart rate variability (HRV), and symptoms of anxiety and depression in women with PCOS.

**Methods:**

Ninety-five women diagnosed with PCOS according to the Rotterdam Consensus criteria were included by convenience sampling. Groups were stratified according to the presence of possible or probable anxiety and depression. Clinical, biochemical, anthropometric parameters, and body composition by dual-energy X-ray absorptiometry were evaluated. Anxiety and depression were assessed using the Hospital Anxiety and Depression Scale. Hemodynamic variables and HRV indices were recorded in the supine and standing positions.

**Results:**

Groups with possible or probable anxiety or depression scores exhibited associations with indices indicating lower HRV. A significant negative correlation was observed between the standard deviation of NN intervals and depression in the supine position and between variance and depression. Additionally, participants with possible or probable anxiety scores showed reduced parasympathetic modulation. No significant differences were observed in body composition parameters between groups.

**Conclusion:**

Symptoms of anxiety and depression in women with PCOS were associated with reduced HRV. These findings suggest a potential link between emotional disturbances and autonomic imbalance in this population, emphasizing the relevance of integrated clinical assessment. Future studies should investigate whether emotional or psychological therapies can improve cardiac autonomic modulation in women with PCOS.

## Introduction

Polycystic ovary syndrome (PCOS) is a multifactorial condition that affects approximately 10% of women of reproductive age.^(1,2)^ This syndrome is associated with significant endocrine and metabolic disturbances, characterized by elevated androgen levels, insulin resistance, and an adverse lipid profile.^(3,4)^ In addition, women with PCOS exhibit cardiovascular autonomic imbalance, evidenced by reduced heart rate variability (HRV) and predominance of sympathetic modulation.^(5-7)^ This autonomic pattern is reflected by increased indices of sympathetic modulation, such as the ratio between low-frequency (LF) and high-frequency (HF) components of HRV, and is closely associated with higher cardiovascular risk.^(8,9)^ Thus, autonomic imbalance may represent an important link between the metabolic disturbances of PCOS and its long-term cardiovascular complications.

Beyond metabolic and autonomic alterations, women with PCOS also experience lower quality of life and a higher prevalence of anxiety and depressive symptoms.^(10)^ These impairments in mental health may be partly related to the disease's impact on body image.^(11,12)^ The excess androgens characteristic of PCOS contribute to the development of hirsutism, acne, and obesity,^(13,14)^ conditions that negatively affect self-esteem and are associated with increased levels of anxiety and depression in this population.^(11,15-19)^

In this context, anxiety and depressive symptoms may also be associated with cardiovascular autonomic imbalance. Indeed, studies in different populations have shown that higher levels of anxiety and depression correlate with lower HRV and greater sympathetic modulation, indicating impaired autonomic cardiac regulation.^(20-24)^ However, despite the relevance of this association, no studies to date have examined whether it is present in women with PCOS.

Therefore, the present study aimed to investigate the association between anxiety and depressive symptoms and cardiac autonomic modulation, assessed by heart rate variability, in women with polycystic ovary syndrome, in order to contribute to the understanding of the mechanisms linking mental health and cardiovascular risk in this population.

## Methods

### Sampling

In total, 95 premenopausal women with PCOS, aged between 18 and 39 years, and with a body mass index (BMI) between 18.0 and 39.9 kg/m^2^ were included. This cross-sectional study used convenience sampling, including all eligible women who attended the Gynecology and Obstetrics Clinic of the Ribeirão Preto Medical School Hospital (HCFMRP/USP) during the recruitment period. Patients were diagnosed with PCOS according to the Rotterdam Consensus criteria (Rotterdam Consensus, 2004). Exclusion criteria included a history of (a) smoking, (b) cognitive disturbances, (c) pregnancy, (d) cardiovascular diseases, and (e) use of any medication, including contraceptives. The study was approved by the Ethics Committee (Protocol No. 9640/2014), and all participants provided written informed consent after being fully informed about the study procedures.

### Protocols

#### Clinical and Biochemical measurements

The concentrations of total testosterone, androstenedione, sex steroid hormone-binding globulin (SHBG) and fasting insulin were determined using a chemiluminescent method (Immulite 1000; Immunoassay System; Siemens®, Santa Ana, CA, USA). Fasting blood glucose was determined using the oxidase method (CMD 800X1 / CMD 800iX1, Wiener Lab, São Paulo, Brazil). Fasting High-density lipoprotein (HDL), total cholesterol, and triglycerides were evaluated using the enzymatic method (CMD 800X1 / CMD 800iX1, Wiener Lab, São Paulo, Brazil). Low-density lipoprotein (LDL) was measured using the Fried Ewald formula: LDL cholesterol = total cholesterol (HDL cholesterol + triglycerides/5). The Free Androgen Index (FAI) was obtained from the following formula: (total testosterone (nmol/L)/SHBG (nmol/L) × 100). The homeostatic model assessment of insulin resistance (HOMA-IR) was evaluated using the formula: (fasting blood glucose in mg/dL 0.05551) fasting insulin µUI/mL/22.5.

#### Anthropometry and DXA

Body composition was assessed with a full body scan using dual energy X-ray absorptiometry (DXA) (Hologic 4500 device QDR Discovery® Series; Hologic, Waltham, MA, USA). Analysis was performed using the 5 Discovery Wi model (SN/84826) software (version 13.0). The regions of interest (ROIs) for assessment of the total mass (TM) [fat mass (g) plus lean mass including bone mineral content (g)] and the percentage fat (%) (fat mass/total mass × 100) were as follows: whole body, head, arms, legs and trunk. Furthermore, android and gynoid fat distributions were calculated. Arm and leg fat (%) were defined as the sum of the fat percentage in both arms and both legs divided by two (mean). Since fat distribution may be as important as total fat mass, the following indices were calculated: the android/gynoid ratio, the fat mass/height^2^ (kg/m^2^) and the percentage of fat in the trunk/percentage of fat in the legs. The lean mass index was also calculated as follows: lean mass/height^2^ (kg/m^2^).^(25)^ The volunteers were instructed to not practice physical activities and/or modify their daily life habits before DXA. Waist circumference (WC) and hip circumference (HC) were obtained based on the ACSM recommendations.^(26)^ The BMI (kg/m^2^) was calculated by dividing body weight by the square of the height, and the waist-to-hip ratio (WHR) was calculated by dividing WC (cm) by HC (cm).

#### Anxiety and Depression

The Hospital Anxiety and Depression Scale (HADS) was used to assess anxiety and depression. This test has 14 items (each scored from 0-3), with 7 items assessing anxiety (HADS-A) and 7 items assessing depression (HADS-D). A HADS-A score of 8 or more was considered positive for anxiety (possible or likely), and a HADS-D score of 8 or more as positive (possible or likely) for depression.^(27)^

#### Hemodynamic assessment

Data on systolic blood pressure (SBP), diastolic blood pressure (DBP), and mean blood pressure (MBP) were obtained using digital plethysmography recording equipment (Finometer Pro, Finapres Medical System, Amsterdam, Netherlands). HR data were obtained using an electrocardiographic (ECG) digital recorder through the CM5 lead (ML866 PowerLab).

#### HRV

Heart rate variability data were obtained using the RR intervals (RRi) from the ECG records at a sampling frequency of 2000 Hz. The data interface to the microcomputer was performed using a PowerLab4/35 device (ADInstruments). The data were recorded and stored (Software LabChart 8.0, ADInstruments), for further analysis. The participants were instructed to remain in the supine position for approximately 10 min to stabilize the cardiovascular parameters. After this period, the ECG signals and arterial pulse pressure were recorded simultaneously for another 10 min, followed by a 10-min recording in the orthostatic position (Tilt Test). The temperature (22°C) and ambient lighting were controlled, and the sessions were performed in a noise-free environment. HRV analyses were performed using CardioSeries v2.4 software (http://sites.google.com/site/cardioseries), employing linear (time- and frequency-domain) and nonlinear (symbolic) methods. Linear methods assume that RRi fluctuations occur in a predictable and proportional manner, whereas nonlinear methods capture the complex, non-linear dynamics of RRi. Therefore, the integrated analysis of variables obtained from linear and nonlinear methods provides a more comprehensive assessment of autonomic modulation. In the time domain, the standard deviation of all normal RR intervals (SD) was used as an index of overall HRV, and the root mean square of successive differences between adjacent RR intervals (RMSSD) was used to reflect parasympathetic modulation. In the frequency domain, spectral analysis (fast Fourier transform, FFT) was used to obtain LF (0.04–0.15 Hz) and HF (0.15–0.40 Hz) oscillations, expressed in both absolute (ms^2^) and normalized units (nu). Absolute LF oscillations reflects both sympathetic and parasympathetic influences, whereas LF in normalized units (LFnu) indicates greater sympathetic predominance. HF oscillations, expressed in either ms^2^ or nu, is used as a marker of parasympathetic modulation. The LF/HF ratio was considered an index of sympathovagal balance. In the symbolic analysis, 0V% (no variation), and 2UV% (two unlike variations) patterns were used to characterize sympathetic and parasympathetic modulation, and the 0V/2V ratio was calculated as an additional index of autonomic balance.^(8,28-31)^

#### Statistical analysis

The Shapiro-Wilk test was used to analyze the distribution of quantitative data, with a significance of 95% in each of the variables analyzed. To compare Clinical and Biochemical Measurements, Anthropometry and DXA, Hemodynamic Assessment and HRV of the groups with anxiety and/or depression versus possible/probable anxiety and/or depression, the t test was used to analyze the parametric data, and the Mann-Whitney U was used for non-parametric data. To analyze the correlation between anxiety and depression with all the variables analyzed, the Pearson test was used for parametric data and Spearman for non-parametric data. Data are presented as means and standard errors of the means (SEMs). The differences were considered significant when p < 0.05. SigmaStat 11.0 software (Systat Software Inc., San Jose, CA, USA) was used for the statistical analysis.

## Results

In tables 1, 2, and 3, the groups were divided for comparison between women with PCOS, according to the score found in the answers to the questionnaire, with unlikely anxiety (n = 45) versus possible and likely anxiety (n = 50) and unlikely depression (n = 59) versus possible and likely depression (n = 36). A general characterization of the sample is also presented. In all cases, data were allocated as mean and standard error of the mean (SE). Table 1 displays data on anthropometric indices, androgens, and metabolic parameters in the sample of women with polycystic ovary syndrome, categorized by anxiety and depression. This table shows that older women with PCOS exhibited higher levels of anxiety compared to younger women (p = 0.045). All other variables analyzed and represented in this table (weight, BMI, waist circumference, hip circumference, WHR, triglycerides, cholesterol, HDL, LDL, body fat percentage, testosterone, androstenedione, SHBG, FAI, fasting glycemia, fasting insulin, and HOMA-IR) showed no significant differences between the analyzed groups.

**Table 1:** Anthropometric indices, androgens and metabolic parameters in the sample of women with polycystic ovary syndrome, according to anxiety and depression symptom categories

Variables		Sample characterization (n=95)	Unlikely anxiety (n=45)	Possible and probable anxiety (n=50)	p-value	Unlikely depression (n=36)	Possible and probable depression (n=59)	p-value
Anthropometric and metabolic parameters								
	Age, years	28.8(0.5)	27.7(0.8)	29.8(0.7)*	0.045	28.2(0.7)	29.7(0.8)	0.212
	Height, m	1.62(0.01)	1.62(0.05)	1.62(0.07)	0.953	1.62(0.06)	1.62(0.06)	0.489
	Weight, Kg	74.7(1.6)	74.6(2.0)	74.8(2.35)	0.823	72.8(1.9)	77.8(2.6)	0.121
	BMI, kg/m^2^	28.5(0.5)	28.5(0.7)	28.4(0.79)	0.863	27.8(0.7)	29.5(0.9)	0.133
	Waist circumference, cm	87.1(1.3)	86.4(1.8)	87.7(1.93)	0.628	85.2(1.6)	90.2(2.3)	0.067
	Hip circumference, cm	106.6(1.0)	106.6(1.3)	106.7(1.5)	0.958	105.3(1.3)	108.8(1.6)	0.089
	WHR	0.81(0.01)	0.81(0.01)	0.82(0.01)	0.502	0.81(0.01)	0.83(0.01)	0.293
	Triglyceride, mg/dL	125(9)	121(12)	129(13)	0.855	116(10)	137(16)	0.326
	Cholesterol, mg/dL	190(4)	189(6)	191(5)	0.665	190(5)	191(6)	0.634
	HDL, mg/dL	50.4(1.3)	52.5(2.0)	48.5(1.5)	0.232	51.6(1.7)	48.8(1.9)	0.390
	LDL, mg/dL	114(3)	112(5)	116(4)	0.272	114(4)	114(4)	0.692
	Body fat percentage, %	40.9(0.5)	41.3(0.8)	40.5(0.7)	0.467	40.6(0.7)	41.9(0.9)	0.260
Androgens and insulin resistance parameters								
	Testosterone, ng/dL	90(5)	86(6)	95(8)	0.626	92(7)	88(7)	0.772
	Androstenedione, ng/dL	92(6)	89(8)	94(8)	0.623	92(7)	92(8)	0.651
	SHBG, nmol/L	52(4)	55(6)	49(4)	1.000	55(5)	48(5)	0.459
	FAI	7.83(0.53)	7.12(0.58)	8.47(0.85)	0.381	7.52(0.62)	8.34(0.96)	0.522
	Fasting glycemia, mg/dL (mg/dL)	86(1)	88(2)	84(2)	0.107	86(1.5)	86(1.9)	0.695
	Fasting insulin, µIU/mL	10.6(0.75)	9.45(0.95)	11.66(1.10)	0.193	9.81(0.95)	11.95(1.20)	0.086
	HOMA-IR	2.27(0.16)	2.08(0.21)	2.43(0.23)	0.299	2.10(0.21)	2.54(0.26)	0.082

Values are expressed as mean ± standard error of the mean. Anxiety and depression were assessed using the Hospital Anxiety and Depression Scale (HADS), with scores ≥8 indicating possible or probable anxiety (HADS-A) or depression (HADS-D). % - percent; BMI - body mass index; cm - centimeter; FAI - free androgen index; HDL - high-density lipoprotein cholesterol; HOMA-IR - homeostasis model assessment of insulin resistance; Kg - kilogram; LDL - low-density lipoprotein cholesterol; mg/dL - milligrams per deciliter; m - meter; ng/dL - nanograms per deciliter; nmol/L - nanomoles per liter; µIU/mL - micro-international units per milliliter; SHBG - sex hormone-binding globulin; WHR - waist-to-hip ratio

* p < 0.05 indicates a significant difference between Unlikely Anxiety and Possible/Probable Anxiety groups

**Table 2:** Hemodynamic parameters and heart rate variability (HRV) in supine position in the sample of women with polycystic ovary syndrome, according to anxiety and depression symptom categories

Variables		Sample characterization (n = 95)	Unlikely anxiety (n = 45)	Possible and probable anxiety (n = 50)	p-value	Unlikely depression (n = 36)	Possible and probable depression (n = 59)	p-value
Hemodynamic parameters								
	HR, bpm	76.2(1.1)	73.4(1.5)	78.4(1.4)*	0.006	74.7(1.4)	78.6(1.6)^#^	0.038
	SBP, mmHg	107(1.1)	106(1.6)	107(1.4)	0.670	106(1.5)	109(1.6)	0.141
	DBP, mmHg	71.2(1.0)	69.6(1.5)	72.9(1.2)	0.094	69.2(1.2)	74.5(1.5)^#^	0.008
	MAP, mmHg	83.0(0.9)	81.9(1.4)	84.3(1.2)	0.181	81.3(1.2)	85.8(1.4)^#^	0.018
HRV - Time domain								
	RRi, ms	868(11)	890(18)	848(14)	0.074	884(16)	842(15)	0.078
	SD	54.4(2.7)	60.7(4.4)	48.7(3.1)	0.068	61.0(3.8)	43.6(3.0)^#^	0.005
	RMSSD, ms	52.2(3.8)	59.6(6.1)	45.6(4.4)	0.143	60.2(5.4)	39.1(4.1)^#^	0.021
HRV – Frequency domain								
	Variance, ms^2^	2895(297)	3584(519)	2276(280)	0.099	3470(426)	1953(306)	0.020
	LF, ms^2^	796(84)	930(138)	675(97)	0.142	920(118)	593(104)	0.106
	HF, ms^2^	1349(184)	1738(330)	999(162)	0.207	1678(270)	809(168)	0.054
	LF, nu	43.7(1.61)	42.5(2.2)	44.7(2.3)	0.511	42.2(2.1)	46.0(2.6)	0.264
	HF, nu	56.3(1.61)	57.5(2.2)	55.3(2.3)	0.511	57.8(2.1)	54.0(2.6)	0.264
	LF/HF Ratio	0.97(0.07)	0.93(0.10)	1.00(0.09)	0.403	0.92(0.09)	1.05(0.12)	0.203
HRV – Symbolic domain								
	0V %	20.0(1.4)	19.1(2.0)	21.2(1.9)	0.342	31.9(12.3)	39.7(18.9)	0.480
	2 LV %	12.2(0.8)	13.6(1.4)	11.7(1.0)	0.371	44.7(32.0)	77.6(66.3)	0.504
	2 UV%	22.0(1.1)	23.7(1.6)	20.3(1.4)	0.107	22.6(1.4)	20.5(1.6)	0.487
	2 V% total	34.1(1.5)	37.3(2.5)	32.0(2.0)	0.096	67.3(32.0)	98.1(66.4)	0.392
	OV/2V Ratio	1.11(0.18)	1.09(0.31)	1.11(0.19)	0.281	1.13(0.26)	1.07(0.22)	0.509

Values are expressed as mean ± standard error of the mean. Anxiety and depression were assessed using the Hospital Anxiety and Depression Scale (HADS), with scores ≥8 indicating possible or probable anxiety (HADS-A) or depression (HADS-D). % - percent; 0V% - percentage of sequences of three consecutive RRi with no significant variations; 2LV% - percentage of sequences of three consecutive RRi with two like variations; 2UV% - percentage of sequences of three consecutive RRi with two unlike variations; 2V% total - total percentage of sequences of three consecutive RRi with two variations; bpm - beats per minute; DBP - diastolic blood pressure; HF - high-frequency oscillations; HR - heart rate; LF - low-frequency oscillations; LF/HF - low-to-high frequency ratio; MAP - mean arterial pressure; mmHg - millimeters of mercury; ms - milliseconds; ms^2^ - square milliseconds; nu - normalized units; OV/2V ratio - ratio between 0V% and 2V% total; RMSSD - root mean square of successive differences; RRi - mean R-R interval; SBP - systolic blood pressure; SD - standard deviation of R-R intervals; Variance - variance of R-R intervals

* p < 0.05 indicates a significant difference between Unlikely Anxiety and Possible/Probable Anxiety groups; #p < 0.05 indicates a significant difference between Unlikely Depression and Possible/Probable Depression groups

**Table 3:** Heart rate variability (HRV) in orthostatic position (after tilt test maneuver) in the sample of women with polycystic ovary syndrome, according to anxiety and depression symptom categories

Variables		Sample characterization (n = 95)	Unlikely anxiety (n = 45)	Possible and probable Anxiety (n = 50)	p-value	Unlikely depression (n = 36)	Possible and probable Depression (n = 59)	p-value
HRV – Time domain								
	RRi, ms	714(9)	721(16)	708(10)	0.688	716(13)	711(11)	0.935
	SD	49.1(1.8)	50.1(2.8)	47.2(2.2)	0.517	51.0(2.4)	45.7(2.4)	0.278
	RMSSD, ms	24.9(1.6)	26.7(2.7)	22.9(1.7)	0.505	26.4(2.2)	21.9(1.8)	0.369
HRV – Frequency domain								
	Variance, ms^2^	2139(158)	2301(258)	1993(183)	0.531	2368(230)	1764(162)	0.164
	LF, ms^2^	777(56)	803(90)	753(68)	0.991	861(79)	640(68)	0.118
	HF, ms^2^	419(66)	504(124)	342(52)	0.426	516(102)	260(38)	0.122
	LF, nu	70.0(1.6)	68.2(2.5)	71.9(2.0)	0.310	68.8(2.1)	72.4(2.3)	0.347
	HF, nu	30.0(1.6)	31.8(2.5)	28.1(2.0)	0.310	31.2(2.1)	27.6(2.3)	0.347
	LF/HF Ratio	3.56(0.30)	3.44(0.48)	3.67(0.38)	0.342	3.46(0.40)	3.73(0.46)	0.347
HRV – Symbolic domain								
	0V %	45.7(1.7)	42.7(2.4)	48.4(2.2)	0.085	44.9(2.2)	47.0(2.4)	0.519
	2 LV %	5.49(0.52)	6.37(0.79)	4.69(0.68)	0.082	5.88(0.68)	4.86(0.86)	0.224
	2 UV%	7.57(0.58)	8.52(0.98)	6.72(0.63)	0.050	7.95(0.82)	6.95(0.75)	0.373
	2 V% total	13.1(1.0)	14.9(1.5)	11.4(1.1)*	0.038	13.8(1.3)	11.8(1.4)	0.313
	OV/2V Ratio	6.50(0.60)	5.61(0.89)	7.30(0.80)	0.061	6.26(0.79)	6.90(0.95)	0.361

Values are expressed as mean ± standard error of the mean. Anxiety and depression were assessed using the Hospital Anxiety and Depression Scale (HADS), with scores ≥8 indicating possible or probable anxiety (HADS-A) or depression (HADS-D). % - percent; 0V% - percentage of sequences of three consecutive RRi with no significant variations; 2LV% - percentage of sequences of three consecutive RRi with two like variations; 2UV% - percentage of sequences of three consecutive RRi with two unlike variations; 2V% total - total percentage of sequences of three consecutive RRi with two variations; bpm - beats per minute; HF - high-frequency oscillations; LF - low-frequency oscillations; LF/HF - low-to-high frequency ratio; mmHg - millimeters of mercury; ms - milliseconds; ms^2^ - square milliseconds; nu - normalized units; OV/2V ratio - ratio between 0V% and 2V% total; RMSSD - root mean square of successive differences; RRi - mean R-R interval; SD - standard deviation of R-R intervals; Variance - variance of R-R intervals

* p < 0.05 indicates a significant difference between Unlikely Anxiety and Possible/Probable Anxiety groups

Table 2 presents data on hemodynamic parameters and HRV in the supine position in the sample of women with PCOS, categorized by anxiety and depression. Women with PCOS with possible/likely anxiety showed higher HR (p = 0.004) than those with unlikely anxiety. Women classified with possible/likely depression had higher HR, DBP, and MBP (p = 0.038, p = 0.008, and p = 0.018, respectively) compared to those with unlikely depression. Additionally, women with possible/likely depression exhibited reduced HRV in the time and frequency domains, with significant reductions in SD, RMSSD, and Variance (p = 0.005, p = 0.021, and p = 0.020, respectively). Other HRV variables analyzed in the time, frequency, and symbolic domains showed no significant differences.

Table 3 analyzes HRV variables in the orthostatic position (after tilt test maneuver) in the sample of women with PCOS, categorized by anxiety and depression. This analysis revealed lower parasympathetic cardiac autonomic modulation, as indicated by reduced total 2V% (p = 0.038) and a trend toward lower 2UV% (p = 0.050) in women with possible/likely anxiety compared to those with unlikely anxiety. Other HRV variables analyzed in the time, frequency, and symbolic domains showed no significant differences.

Tables 4 and 5 respectively present data on the correlation of anthropometric indices, androgens, and metabolic parameters with anxiety and depression in the sample of women with PCOS and the correlation of hemodynamic parameters and HRV in the supine and orthostatic positions with anxiety and depression in the same group. A significant negative correlation was observed between the SD variable and depression (R = -0.238; p = 0.021) in the supine position and between variance and depression (R = -0.203; p = 0.049). No correlations were identified for other analyzed variables. Other variables (age, weight, BMI, waist circumference, hip circumference, WHR, triglycerides, cholesterol, HDL, LDL, body fat percentage, testosterone, androstenedione, SHBG, FAI, fasting glycemia, fasting insulin, and HOMA-IR) and HRV variables analyzed in the time, frequency, and symbolic domains showed no significant correlations.

**Table 4:** Correlation of anthropometric indices, androgens and metabolic parameters with anxiety and depression symptoms in women with polycystic ovary syndrome

Variables		Anxiety		Depression	
		R	p	R	P
Anthropometric and metabolic parameters					
	Age, years	0.111	0.283	0.057	0.582
	Height, m	-0.056	0.587	-0.003	0.971
	Weight, Kg	-0.027	0.797	0.128	0.217
	BMI, kg/m^2^	-0.009	0.927	0.134	0.194
	Waist circumference, cm	-0.026	0.802	0.068	0.515
	Hip circumference, cm	0.031	0.765	0.167	0.106
	WHR	-0.027	0.791	-0.011	0.917
	Triglyceride, mg/dL	0.107	0.301	0.159	0.124
	Cholesterol, mg/dL	0.191	0.063	0.118	0.256
	HDL, mg/dL	0.018	0.864	0.058	0.578
	LDL, mg/dL	0.185	0.075	0.074	0.478
	Body Fat Percentage, %	0.021	0.840	0.168	0.103
Androgens and insulin resistance parameters					
	Testosterone, ng/dL	0.028	0.788	-0.026	0.804
	Androstenedione, ng/dL	0.090	0.386	0.144	0.165
	SHBG, nmol/L	-0.045	0.664	-0.223	0.314
	FAI	0.121	0.241	0.046	0.656
	Fasting Glycemia, mg/dL	-0.045	0.664	-0.035	0.735
	Fasting Insulin, µIU/mL	0.104	0.317	0.093	0.368
	HOMA-IR	0.088	0.393	0.109	0.292

Values are expressed as correlation coefficients (R) and respective p values. Anxiety and depression were assessed using the Hospital Anxiety and Depression Scale (HADS), with scores ≥8 indicating possible or probable anxiety (HADS-A) or depression (HADS-D). % - percent; BMI - body mass index; cm - centimeters; FAI - free androgen index; HDL - high-density lipoprotein cholesterol; HOMA-IR - homeostasis model assessment of insulin resistance; kg - kilograms; LDL - low-density lipoprotein cholesterol; m - meters; mg/dL - milligrams per deciliter; ng/dL - nanograms per deciliter; nmol/L - nanomoles per liter; SHBG - sex hormone-binding globulin; µIU/mL - micro-international units per milliliter; WHR - waist-to-hip ratio; years - years of age

**Table 5:** Correlation of hemodynamic parameters and heart rate variability (HRV) in supine position and orthostatic position (after tilt test maneuver) with anxiety and depression symptoms in women with polycystic ovary syndrome

Variables		Anxiety		Depression	
		R	p	R	P
Hemodynamic parameters					
	HR, bpm	0.036	0.743	0.113	0.302
	SBP, mmHg	-0.076	0.490	0.028	0.795
	DBP, mmgHg	0.073	0.505	0.036	0.741
	MAP, mmHg	0.013	0.950	0.025	0.820
HRV - Time domain (supine)					
	RRi, ms	-0.121	0.243	-0.123	0.234
	SD	-0.183	0.076	-0.238	0.021
	RMSSD, ms	-0.127	0.220	-0.166	0.108
HRV – Frequency domain (supine)					
	Variance, ms^2^	-0.150	0.146	-0.183	0.076
	LF, ms^2^	-0.154	0.135	-0.127	0.219
	HF, ms^2^	-0.105	0.308	-0.140	0.176
	LF, nu	0.030	0.774	0.027	0.792
	HF, nu	0.030	0.774	-0.027	0.792
	LF/HF Ratio	0.030	0.774	-0.027	0.792
HRV – Symbolic domain (supine)					
	0V %	-0.033	0.748	-0.136	0.189
	2 LV %	0.069	0.504	0.141	0.172
	2 UV%	-0.027	0.790	0.096	0.352
	2 V% total	-0.029	0.781	0.114	0.272
	OV/2V Ratio	-0.029	0.781	-0.131	0.206
HRV - Time domain (orthostatic)					
	RRi, ms	-0.038	0.720	-0.012	0.907
	SD	-0.182	0.081	-0.201	0.053
	RMSSD, ms	-0.087	0.407	-0.113	0.280
HRV – Frequency domain (orthostatic)					
	Variance, ms^2^	-0.127	0.221	-0.203	0.049
	LF, ms^2^	-0.061	0.556	-0.198	0.054
	HF, ms^2^	-0.072	0.486	-0.164	0.112
	LF, nu	0.037	0.723	0.072	0.490
	HF, nu	-0.037	0.723	0.072	0.490
	LF/HF Ratio	-0.037	0.723	0.072	0.490
HRV – Symbolic domain (orthostatic)					
	0V %	0.049	0.638	-0.104	0.316
	2 LV %	-0.048	0.642	0.068	0.510
	2 UV%	-0.045	0.664	0.076	0.462
	2 V% total	-0.052	0.612	0.092	0.375
	OV/2V Ratio	0.046	0.657	-0.096	0.352

Values are expressed as correlation coefficients (R) and respective p values. Anxiety and depression were assessed using the Hospital Anxiety and Depression Scale (HADS), with scores ≥8 indicating possible or probable anxiety (HADS-A) or depression (HADS-D). Abbreviations: % - percent; 0V% - percentage of sequences of three consecutive RRi with no significant variations; 2LV% - percentage of sequences of three consecutive RRi with two like variations; 2UV% - percentage of sequences of three consecutive RRi with two unlike variations; 2V% total - total percentage of sequences of three consecutive RRi with two variations; bpm - beats per minute; DBP - diastolic blood pressure; HF - high-frequency oscillations; HR - heart rate; LF - low-frequency oscillations; LF/HF - low-to-high frequency ratio; MAP - mean arterial pressure; mmHg - millimeters of mercury; ms - milliseconds; ms^2^ - square milliseconds; nu - normalized units; OV/2V ratio - ratio between 0V% and 2V% total; RMSSD - root mean square of successive differences; RRi - mean R-R interval; SBP - systolic blood pressure; SD - standard deviation of R-R intervals; Variance - variance of R-R intervals

## Discussion

This study identified that 50 out of the 95 women evaluated (52.6%) presented possible or probable anxiety behavior, while 36 out of the 95 (37.9%) had possible or probable depression. These numbers represent a relatively high rate of anxiety and depression in this group of women. We also assessed whether there were differences in anthropometric indices, androgens, and metabolic parameters among women with PCOS with unlikely anxiety scores versus possible or probable anxiety, as well as among women with unlikely depression scores versus possible or probable depression. Additionally, HRV was compared in supine and standing positions in these groups, along with a correlation analysis involving all HRV variables and the anxiety and depression scores. It was possible to identify differences in age, where older age was associated with higher levels of anxiety, as well as lower HRV and increased sympathetic cardiac autonomic modulation in groups with higher anxiety or depression. No differences or correlations were identified in the other endocrine, metabolic, and body composition parameters evaluated.

Previous studies had already evidenced increased anxiety and depression in women with PCOS.^(15,19)^ Authors have suggested that the increased rates of anxiety and depression in women with PCOS may be related to various factors, including dissatisfaction with body image.^(17-19)^ Although we did not find differences or associations between the groups regarding endocrine and metabolic parameters, one study made an association, pointing out that women with PCOS with higher anxiety and depression scores had hyperandrogenism and the presence of oligoovulation.^(32)^ Researchers associated anxiety with age and hyperandrogenism and depression with age, hyperandrogenism, and metabolic factors, including fasting insulin, fasting plasma glucose, and HOMA-IR.^(19)^ On the other hand, another search, when comparing a sample of 478 women with PCOS and 8,134 without PCOS, despite mentioning findings related to increased depression, anxiety, and perceived stress in women with PCOS, suggested that further studies were needed to better understand the etiology of these conditions.^(15)^

Given this, we sought to understand whether other parameters, such as cardiac autonomic modulation, could interfere with anxiety and depression since impairments in cardiac autonomic modulation in women with PCOS had already been identified in other studies.^(6,33,34)^ In a study with 64 women, it was possible to observe greater sympathetic cardiac autonomic modulation in women with PCOS compared to the control group after a provocative test with a change from the supine to the upright position (tilt test), a behavior that persisted upon returning to the upright posture.^(6)^ The finding of sympathetic cardiac hyperactivity in women with PCOS has also been reported in other studies, including with reduced parasympathetic modulation and HRV.^(6,33,34)^ Thus, this alteration motivated us to try to understand whether, in women with PCOS, it could be associated with changes in anxiety and depression scores, as associations between these variables have been reported in other populations but not yet in PCOS.^(24,35)^

Some researchers evidenced, when evaluating 265 subjects, that those with a history of major depression showed dysregulation of cardiac autonomic modulation, significantly reducing respiratory sinus arrhythmia compared to the control group.^(24)^ In adolescents with depression, regulatory changes related to the heart were identified through a nonlinear symbolic analysis.^(35)^ Additionally, corroborating these results, in one study it was observed an association between depression and cardiac autonomic modulation, particularly with reduced parasympathetic modulation and increased sympathetic activity.^(36)^ This association involving HRV was also observed in middle-aged individuals with increased anxiety and depressive symptoms, displaying an unfavorable cardiac autonomic profile.^(37)^

Regarding our findings, we identified differences indicating altered cardiac autonomic modulation in women with PCOS presenting anxiety and depression symptoms. In the supine position, women with possible or probable anxiety showed higher heart rate, whereas those with possible or probable depression exhibited higher heart rate, diastolic, and mean blood pressure, as well as reduced HRV, with lower SD and RMSSD in the time domain compared to those without depressive symptoms. When the position changed to upright, women with possible or probable anxiety presented lower values of 2UV% and total 2V%, variables associated with parasympathetic modulation. These differences between the supine and upright positions likely reflect physiological adjustments in autonomic balance. In the supine position, venous return is facilitated and parasympathetic tone predominates, resulting in a lower heart rate. Upon assuming the upright posture, venous return decreases, initially accompanied by parasympathetic withdrawal followed by increased sympathetic tone to maintain blood pressure.^(38,39)^ In women with anxiety, basal sympathetic hyperactivity or heightened stress reactivity may amplify this parasympathetic withdrawal, potentially explaining the greater reduction in 2UV% and total 2V% observed during orthostasis. Further studies are needed to test this hypothesis and elucidate the mechanisms linking emotional state to cardiac autonomic imbalance.

Numerous studies on cardiac autonomic modulation have already shown an increased risk of developing cardiovascular diseases when HRV is reduced, with imbalances in cardiac autonomic modulation leading to sympathetic hyperactivity and decreased parasympathetic activity.^(24,33,40)^ Over time, this imbalance, associated with other changes such as anxiety and depression, may exacerbate the likelihood of developing cardiovascular and emotional disorders.^(24,40)^

Although our findings indicate associations between anxiety and depression symptoms and HRV parameters, these results should be interpreted with caution. Heart rate and blood pressure are influenced by multiple transient factors, including white-coat effects, momentary nervousness, and environmental conditions. As this was a single assessment, we cannot infer causality or propose HRV as a definitive marker of anxiety or depression in women with PCOS, or vice versa. Longitudinal studies with repeated measurements and comprehensive evaluation of psychiatric history are needed to confirm these relationships. Moreover, while this study did not aim to evaluate therapeutic interventions, existing evidence supports professionally guided strategies - such as structured physical exercise, nutritional approaches, and cognitive-behavioral therapies - to help prevent or mitigate alterations in the variables investigated here.^(41-43)^

We consider the study's strength to be the first to relate cardiac autonomic modulation and symptoms of anxiety and depression in women with PCOS, issues commonly altered in this population and requiring attention and screening. As these variables are related, we suggest that they be clinically evaluated together, including when there are comparisons before and after clinical interventions, with the aim of understanding whether these relationships are maintained and consequently verifying the benefits for women with PCOS.

As a limitation, there are reports that other metabolic and endocrine parameters may contribute to changes in both emotional state parameters and cardiac autonomic modulation, although we did not find evidence in our results. It is worth noting that our sample may have been limited due to heterogeneity in terms of age (18–39 years) and BMI (18.0–39.9 kg/m^2^), factors that might have influenced the analysis of these parameters. We suggest future studies expand investigations, better stratifying groups by age, weight, and BMI, and seeking to understand the breadth of treatment and therapy effects on emotional state and cardiac autonomic modulation simultaneously.

## Conclusion

From our findings, we identified some associations between emotional state and cardiac autonomic modulation in women with PCOS. Groups with possible or probable anxiety or depression scores exhibited associations with indices indicating lower HRV. Additionally, lower parasympathetic modulation was identified in those with possible or probable anxiety scores. Once this relationship was identified, we suggest that when screening cardiac autonomic modulation, negative changes may indicate adverse consequences for emotional state and vice-versa, requiring prevention, care, and treatment in both cases. We recommend that PCOS patients with negative changes and/or repercussions in cardiac autonomic modulation and emotional state seek professionals who employ proven interventions such as physical exercise, cognitive-behavioral therapies, and improved nutrition.

## Data Availability

The research data are described in the article presented.
